# O054: Surgical site infections (SSI) and risk factors in breast cancer surgery (BCS): French survey surviso 2011

**DOI:** 10.1186/2047-2994-2-S1-O54

**Published:** 2013-06-20

**Authors:** M-Y Louis, F Lemarie, P Berger, B Clarisse

**Affiliations:** 1 Surgeon, Head of Hygiene Hospital Department, France; 2 Hygiene Hospital Department, Centre françois Baclesse, Caen, France; 3 Institut Paoli Calmettes, Marseille, France; 4 Clinical Research Department, Centre françois Baclesse, Caen, France

## Introduction

There are sparse data on SSI in oncology that is more frequent than in general population. BCS is a clean surgery performed in patients without co-morbidity. A prospective survey we conducted in 2008 revealed a 4.1% SSI incidence rate in 1686 BCS, resulting in new guidelines.

## Objectives

We aimed to assess the SSI incidence rate in BCS and to identify some risk factors.

## Methods

Data collection concerned BCS, classified I or II in the Altemeier classification, associated or not to immediate breast reconstruction. The survey concerned at least 100 consecutive BCS performed in each of the 15 participating comprehensive cancer centres in the first semester of 2011. Data collection was based on the French SSI reporting (age, ASA and NNISS scores, surgery and SSI dates, involved strain). It was completed with some data specific to cancer setting.

## Results

Data were collected for 2883 BCS, including 2766 initial BCS. The kind of surgery was available for 2731 initial BCS: 1527 (56%) lumpectomies, 563 (21%) mastectomies, 143 (5%) and 170 (6%) immediate and secondary reconstructions, respectively, 35 (1%) node dissections, 293 (11%) breast mammoplasty surgeries.

The SSI incidence rate (median onset delay: 16 days) was 2.86%[CI95%: 2.27-3.55] (79 SSI) as compared to 4.1%[CI95%: 3.20-5.15] in 2008, corresponding to a 30% decrease. *S. aureus* was identified in 58 cases.

The multivariate analysis highlighted several factors related to the risk of SSI onset. A 3-4 ASA score (vs ASA 1) was associated to an adjusted odds ratio (ORa) of 2.51[1.21-5.18]). As compared to lumpectomies without node dissection and prophylactic antibiotics, immediate reconstructions were related to an ORa of 3.65[1.41- 9.42], node dissection without prophylactic antibiotics and associated or not to lumpectomy to an ORa of 4.58[2.13-9.87]. Hematoma and lymphocele punctures were respectively related to an ORa of 3.19[1.33-7.66] and 2.98[1.84-4.83]. No relation was noted for prior chemo/radiotherapy and for invasive preoperative procedures.

## Conclusion

The identification of risk factors specific to cancer setting argued to a particular attention, notably concerning postoperative procedures and surgery techniques.

## Disclosure of interest

None declared.

